# TRPM2 ion channel promotes gastric cancer migration, invasion and tumor growth through the AKT signaling pathway

**DOI:** 10.1038/s41598-019-40330-1

**Published:** 2019-03-12

**Authors:** Shekoufeh Almasi, Andra M. Sterea, Wasundara Fernando, Derek R. Clements, Paola Marcato, David W. Hoskin, Shashi Gujar, Yassine El Hiani

**Affiliations:** 1 Departments of Biology, Dalhousie University, Halifax, Nova Scotia B3H 4R2 Canada; 2 Departments of Physiology, and Biophysics, Dalhousie University, Halifax, Nova Scotia B3H 4R2 Canada; 3 Department of Microbiology and Immunology, Stanford University, Stanford, California 94305 USA; 4 Departments of Pathology, Dalhousie University, Halifax, Nova Scotia B3H 4R2 Canada; 5 Microbiology and Immunobiology, Faculty of Medical Sciences, Dalhousie University, Halifax, Nova Scotia B3H 4R2 Canada

## Abstract

Transient Receptor Potential Melastatin-2 (TRPM2) ion channel is emerging as a great therapeutic target in many types of cancer, including gastric cancer – a major health threat of cancer related-death worldwide. Our previous study demonstrated the critical role of TRPM2 in gastric cancer cells bioenergetics and survival; however, its role in gastric cancer metastasis, the major cause of patient death, remains unknown. Here, using molecular and functional assays, we demonstrate that TRPM2 downregulation significantly inhibits the migration and invasion abilities of gastric cancer cells, with a significant reversion in the expression level of metastatic markers. These effects were concomitant with decreased Akt and increased PTEN activities. Finally, TRPM2 silencing resulted in deregulation of metastatic markers and abolished the tumor growth ability of AGS gastric cancer cells in NOD/SCID mice. Taken together, our results provide compelling evidence on the important function of TRPM2 in the modulation of gastric cancer cell invasion likely through controlling the PTEN/Akt pathway.

## Introduction

Gastric cancer (GC) is one of the most aggressive types of cancer with a significant participation in cancer-related mortality worldwide. H-pylori infection, inappropriate dietary plans, poor sanitation, and smoking are the common risk factors^1^. However, late diagnosis of the disease and metastasis spreading of gastric tumors remain the main reasons for GC mortality^2^. This makes understanding the basic cellular and molecular mechanisms of GC metastasis of high priorities towards the development of new clinical approaches to improve GC therapy.

Longstanding investigations have demonstrated the central role for Akt pathway in the regulation of numerous cellular phenotypes associated with cancer metastasis including migration, invasion and the epithelial-mesenchymal transition (EMT) processes^3–6^. Among several upstream regulators of Akt pathway, PTEN (phosphatase and tensin homolog)^7,8^ and cytosolic calcium homeostasis^9–12^ have been shown to play major roles. PTEN function as a phosphatidyl inositol triphosphate (PIP3) phosphatase, opposing the activity of phosphatidylinositol-3-kinase (PI3K) and negatively regulates Akt^13,14^. Calcium is a universal second messenger with a key role in regulating the Akt pathway^15^ and calcium signaling have been shown involved in critical steps that favour the spread of tumor cells such as the EMT processes^16^. However, the cellular basis and the underlying regulatory mechanisms by which cancer metastasis occur have not been fully documented.

We recently described the calcium-permeable Transient Receptor Potential Melastatin-2 (TRPM2) channel as a prognsostic marker in a cohort of GC patients and demonstrated its role in the bioenergetics and survival of GC cell lines^17^. Here, we further investigate whether TRPM2 holds an important role in GC cells migration and invasion. We demonstrated that TRPM2 contribute to the invasion and metastasis of GC via Akt-mediated EMT, and suggested TRPM2 inhibition as a potential therapeutic approach to hamper GC metastasis and improve GC treatment.

## Results

### TRPM2 activation elicits cytosolic calcium elevation in AGS cells

**Figure 1:**
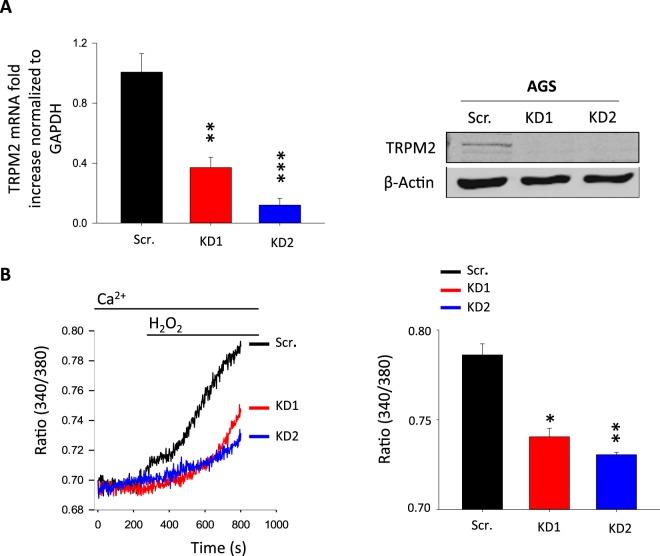
TRPM2 is functionally expressed as a calcium channel in AGS gastric cancer cells. (**A**) Western blot and RT-qPCR analyses of TRPM2 expression in both, AGS scramble and TRPM2-KD cells. (**B**) Calcium imaging analysis of TRPM2 ion channel in AGS scramble and TRPM2-KD cells. 1 mM H_2_O_2_ treatment increased the cytosolic Ca^2+^ level in scramble cells while this effect is significantly decreased in TRPM2-KD cells. Quantification of intracellular Ca^2+^ peak values is expressed as mean ± *SD* and represented as a bar graph. (experiments have been done in triplicate and data are an average of three experiments, *t*-test vs. Scr. ***p < 0.001; **p < 0.01; *p < 0.05).

TRPM2 is identified as a non-selective cation channel, permeable to calcium^18^. We recently demonstrated the functional expression of TRPM2 as a plasma membrane ion channel in GC cells^17^. Here, we extended our investigation to the role of TRPM2 in regulating intracellular calcium ([Ca^2+^]_i_) levels. In the absence of specific inhibitors, the lentiviral-shRNA technique was used to generate two AGS cells in which TRPM2 was knocked down permanently (KD1 and KD2), and the knockdown efficacy was examined using RT-qPCR and western blot analyses (Fig. 1A). Given that TRPM2 is considered as the main sensor of oxidative-stress^19–22^, we have used H_2_O_2_ to stimulate TRPM2-mediated calcium entry^23–25^, and monitored changes in cytoplasmic calcium using calcium imaging method. As well known, the high concentrations of H_2_O_2_ are toxic to human cells^26^; hence, we have used 1 mM of H_2_O_2_ with the minimum cytotoxicity to AGS cells under our experimental conditions. As expected, H_2_O_2_ perfusion induced a significant elevation in [Ca^2+^]_i_ in scrambled AGS cells. This increase in [Ca^2+^]_i_ was significantly reduced in TRPM2-KD cells (Fig. 1B). These data indicate the functional expression of TRPM2 as a calcium channel in AGS cells.

### Genetic silencing of TRPM2 inhibits migration and invasion abilities of AGS cells

**Figure 2:**
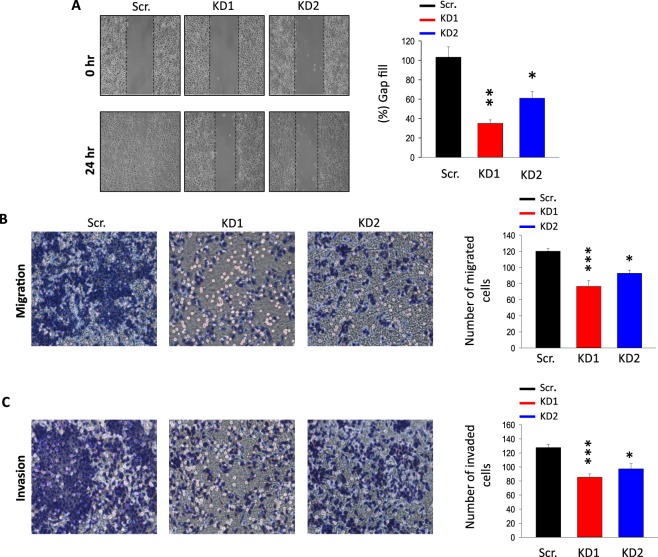
TRPM2 downregulation inhibits the migration and invasion abilities of AGS gastric cancer cells. (**A**) Gap closure migration assay of AGS scramble and TRPM2-KD cells. Data were recorded at the 0-time point and 24 hours later; results are presented as a bar graph. Quantification of cell motility is expressed as mean ± *SD* and represented as a bar graph. (**B**,**C**) Migration and invasion assays of AGS scramble and TRPM2-KD cells. Numbers of migrated and invaded cells were analyzed 24 hours after cells have been seeded in the chamber and data were summarized as bar graphs. The data are represented as the mean of three independent experiments (*t*-test vs. Scr. ***p < 0.001; **p < 0.01; *p < 0,05).

**Figure 3:**
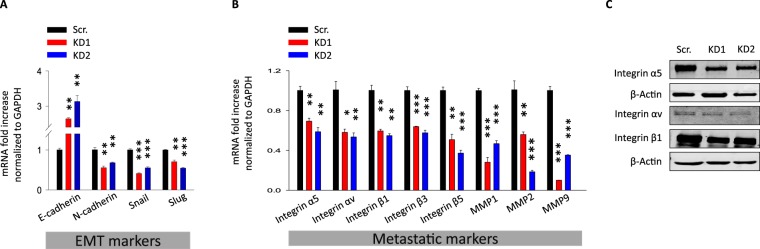
Effect of TRPM2 silencing the expression of the EMT in AGS gastric cancer cells. (**A**,**B**) RT-qPCR analysis of EMT markers, integrins and MMPs in both AGS control and TRPM2-KD cells (RT-qPCR was done in triplicate, *t*-test vs. scr. ***p < 0.001; **p < 0.01; *p < 0.05). (**C**) Cell lysates from AGS cells expressing either control ShRNA, or ShRNA-TRPM2 were analysed by immunoblotting for the endogenous expression of integrins.

We previously have demonstrated the important role of TRPM2 in the survival and the bioenergetics of AGS cells^17^. To evaluate the potential role of TRPM2 in the migration and invasion abilities of GC cells, a gap closure assay was conducted to compare the motility of AGS scramble and TRPM2-KD cells. The results showed a significant reduction in the speed of gap filling in TRPM2-KD cells, suggesting a critical role of TRPM2 in the regulation of GC metastasis (Fig. 2A). Therefore, we have investigated the TRPM2 involvement in AGS cell migration and invasion. Our results showed that TRPM2-KD cells exhibited lower migration and invasion capabilities in comparison to the scramble cells. Indeed, the number of the migrated and invaded TRPM2-KD cells in multi-well chemotaxis chamber assay was significantly less than scramble cells (Fig. 2B,C). On the other hand, TRPM2 silencing led to a significant decrease in the expression level of Epithelial-Mesenchymal Transition (EMT), migration and invasion markers such as N-cadherin, snail, slug, integrins, and MMPs (Fig. 3), suggesting the reduced ability of these cells to migrate and invade to the other tissues. Collectively, these data indicate that TRPM2 contribute to the migration and invasion capabilities of GC cell lines.

### PTEN/Akt signaling pathway is required for TRPM2-mediated migration and invasion abilities of AGS cells

**Figure 4:**
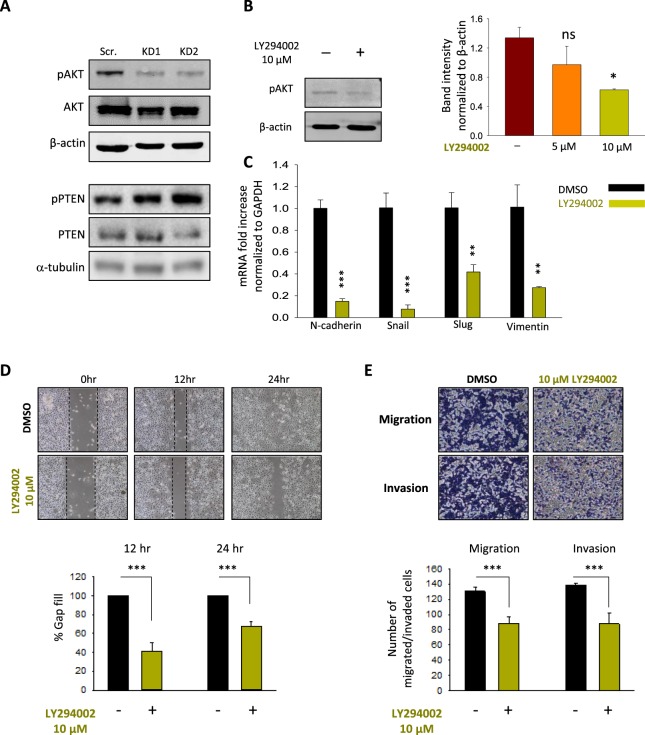
TRPM2 promotes cell migration and invasion through Akt-mediated EMT. (**A**) Western blot analysis of the protein level of phospho-Akt (Ser473), total Akt, phospho-PTEN (Ser380/Thr382/383) and total PTEN in AGS control and TRPM2-KD cells. (**B**) The protein level of phohpho-Akt before and 24 hours after treatment with 10 μM LY294002, a PI3K inhibitor (**C**) mRNA level of EMT markers before and 24 hours after Akt inhibition by LY294002 (**D**) Gap closure assay study of AGS wildtype cells at 0, 12 and 24 hours after LY294002 treatment (10 μM). The average results of three independent experiments were summarized in the corresponding bar graph. (**E**) *In vitro* analysis of migration and invasion ability of AGS cells with or without LY294002 treatment (10 μM) after 24 hrs; the number of migrated and invaded cells from three independent experiments are presented in bar graphs (*t*-test vs. Scr. ***p < 0.001; **p < 0.01; *p < 0.05).

**Figure 5:**
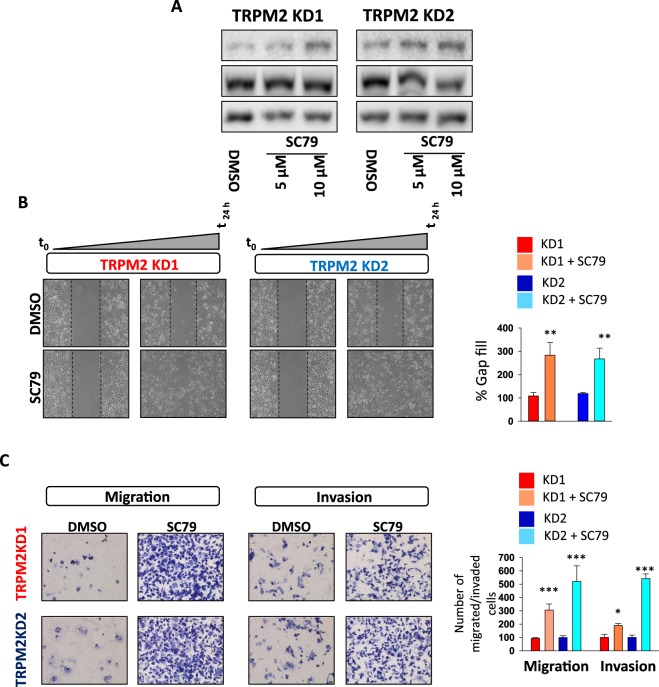
Akt activation rescued the migration and invasions abilities of TRPM2 depleted gastric cancer cells. (**A**) The protein level of phohpho-Akt (Ser473) before and 24 hours after treatment with Akt activator SC79 in AGS scramble and TRPM2-KD cells. (**B**) Gap closure assay study of TRPM2 depleted KD 1 and KD2 cells at 0 and 24 hours after SC79 treatment (10 μM). The average results of three independent experiments were summarized in the corresponding bar graph. (**C**) *In vitro* analysis of migration and invasion ability of TRPM2 depleted AGS cells with or without SC69 treatment (10 μM) after 24 hrs; number of migrated and invaded cells from three independent experiments are presented in bar graphs (*t*-test vs. Scr. ***p < 0.001; **p < 0.01; *p < 0.05).

It is widely recognized that Akt signaling is involved in the regulation of migration and invasion of various tumors, including gastric tumors^27,28^. In order to explore the involved signaling pathways in the TRPM2-mediated control of migration and invasion abilities of GC cells, the activation of Akt signaling was compared between scramble and TRPM2-KD AGS cells. As shown in Fig. 4A, the phosphorylation of Akt at Ser473 was markedly suppressed in TRPM2-KD cells, while the total Akt remained unchanged. This result was concomitant with an increase in the protein level of phospho-PTEN, the direct upstream regulator of Akt, without a change in the level of total PTEN, suggesting the involvement of PTEN/Akt pathway in TRPM2 mediated GC migration and invasion. To investigate the effect of Akt pathway on TRPM2-mediated motility and invasion, LY294002 (10 μM) was used to specifically inhibit the activation of the Akt in wildtype cells^29,30^. Our results demonstrated that 24 hours treatment of AGS scramble cells with 10 μM LY294002 decreases migration and invasion abilities of these cells in both gap closure and multi-well chemotaxis chamber assays, supporting the hypothesis that Akt pathway is required for the TRPM2-mediated motility and invasion of AGS cells (Fig. 4D,E). These results were concomitant with a significant decline in the expression of the EMT markers in the presence of Akt pathway inhibitor LY294002 (Fig. 4C), suggesting the involvement of Akt pathway activation in the TRPM2-mediated upregulation of the EMT markers. To further investigate Akt involvement in the TRPM2-mediated GC cell invasion, AGS cells in which TRPM2 was downregulated were treated with SC79 Akt activator^31^. As shown in Fig. 5, the treatment of TRPM2 KD AGS cells with the Akt-inducer SC79 caused an increase in Akt phosphorylation (Fig. 5), rescued cell motility (Fig. 5), and restored the migration and invasion capabilities of these cells (Fig. 5). Furthermore, SC79 treatment significantly upregulated EMT mRNA markers (unshown data). Taken together, these results indicate that TRPM2-mediated GC cell migration and invasion is likely by controlling the EMT processes through the control of PTEN/Akt signaling pathway.

### TRPM2 silencing inhibits tumor formation ability of AGS cells in NOD/SCID mice

**Figure 6:**
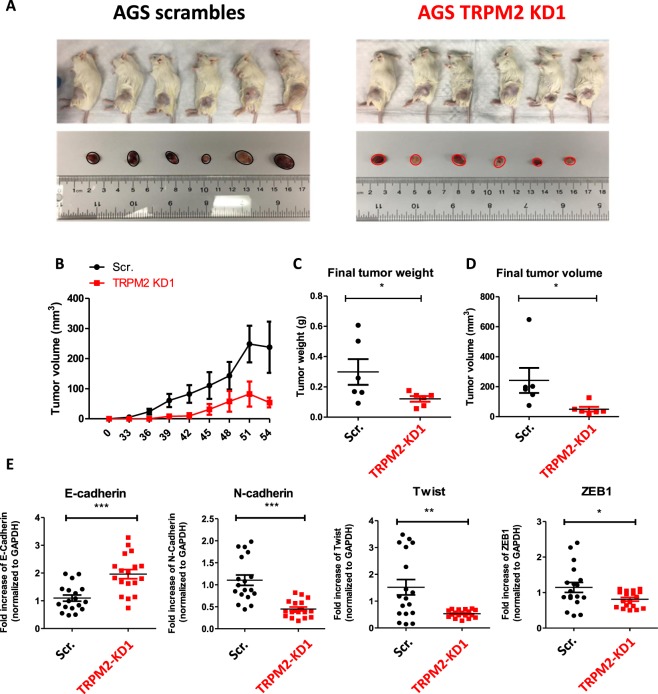
TRPM2 downregulation inhibits GC tumor growth and reverses EMT process in SCID mice. (**A**) Schematic presentation of scrambled and TRPM2-KD AGS tumors in male NOD/SCID mice. Mice were subcutaneously injected with 4 million cells in the left flank. Tumor size was measured every 3 days, 2 weeks post-injection, for 1.5 months. Resulted data are presented in corresponding graphs (**B**) Change in tumor volume for three weeks post-injection. (**C**,**D**) Final tumors’ weight and volume. (**E**–**H**) RT-qPCR analysis of the mRNA expression level of EMT markers in extracted tumors (*t*-test vs. Scr. ***p < 0.001; **p < 0.01; *p < 0.05).

To validate our *in vitro* data on the role of TRPM2 in AGS cell growth and invasion, we investigated the *in vivo* impact of TRPM2 silencing on the AGS tumor xenograft growth in NOD/SCID mice^32^. Male NOD/SCID mice were injected in the left flank with either scramble or TRPM2-KD AGS cells; tumor size was measured twice weekly for six weeks. Our *in vivo* data demonstrated the negative impact of TRPM2 depletion on the tumor growth ability of AGS cells, as reflected by the apparent differences in size between scrambled and TRPM2-KD tumors (Fig. 6A). Indeed, tumors formed by TRPM2-KD cells were significantly smaller and lighter in comparison to scrambled tumors (Fig. 6B–D). Furthermore, RT-qPCR analysis of mRNA samples extracted from both, scramble and TRPM2-KD tumors verified that the expression level of many EMT markers drastically altered in TRPM2-KD tumors. Indeed, the expression of an epithelial marker, E-cadherin increased, while the mRNA level of mesenchymal markers (N-cadherin, Twist, Zeb1, Vimentin, and Slug) significantly decreased in TRPM2-KD tumors compared with scramble tumors (Fig. 6E–J). Overall, these data indicate that TRPM2 downregulation inhibits *in vivo* gastric tumor growth and reverses the EMT process which further confirmed our findings on the role of a TRPM2 ion channel in GC progression.

## Discussion

GC counts as the fifth most prevalent cancer worldwide and causes about 700,000 deaths per year^33^. The low patient survival is mostly due to the late diagnosis of cancer at the metastasis stage. As known, tumor metastasis limits the efficacy of available cancer therapies, suggesting the necessity of discovering new therapeutic approaches^34^. In the last two decades, ion channels gained considerable attention in cancer therapy, and have been used as both, molecular biomarker and therapeutic targets in various types of cancer^35^.

Among many ion channels, TRPM2 is emerging as a new potential therapeutic target in controlling cancer progression^36^. To date, the impact of TRPM2 activation on various signaling pathways in cancer cells survival has been studied^25,37–42^. We have previously demonstrated the functional expression of TRPM2 in GC bioenergetics and survival^17^. Here, we further show its role in controlling GC tumor progression and *in vitro* cell metastasis. Our results indicated that TRPM2 is functionally expressed in GC cell lines to control cytosolic calcium homeostasis and to regulate PTEN/Akt pathway which, in turn, control the EMT process and, thus, cell motility and invasion. In fact, TRPM2 silencing in AGS GC cells led to the deactivation of Akt through upregulation of PTEN, and the reduction in phosphor-Akt level was associated with alteration of both migration and invasion. This is consistent with many previous studies that demonstrated the importance of PTEN/Akt in cancer cell metastasis, and the recognition that drugs targeting Akt function may have great clinical potential as a potent anti-cancer agents^27,43,44^. Indeed, PTEN was found to be downregulated in gastric tumours and its expression profile is related to GC stages, where loss of PTEN expression is highly correlated with advanced stage of GC^45^. Among different mechanisms controlling PTEN activity in GC cells and tissues, the level of PTEN phosphorylation on Ser380 or a decrease in de-phosphorylation of the protein were identified as dominant regulators^46^. It is possible that a similar mechanism activated in AGS cells upon TRPM2 depletion enhances PTEN phosphorylation or reduces its proteasomal degradation^47,48^. Altogether, these data indicate a critical role of TRPM2 in GC cells motility and invasion, likely through the regulation of the PTEN/Akt pathway.

TRPM2-mediated GC migration/invasion was also found associated with an alteration in the regulation of EMT, an essential process during cancer metastasis^49–52^. Strikingly, we find that TRPM2 silencing significantly reversed the expression of EMT markers such as E-cadherin, N-cadherin, snail, and Twist. These data are consistent with previous studies demonstrating the key role of the EMT processes in cancer cells migration and invasion^53^. For instance, the downregulation of Slug has been shown to inhibit tumor metastasis in many cancer types^54,55^, including GC^56^. Furthermore, our *in vivo* experiments revealed that TRPM2 depleted AGS tumors have reduced growth in comparison to AGS control tumors which further confirmed our *in vitro* data. Stingingly, analysis of EMT expression from TRPM2 depleted GC tumors revealed the inhibitory impact of TRPM2 silencing of on the EMT processes, suggesting its key role in GC tumor invasion and metastasis through the induction if the EMT. However, one major limitation of our study is that only one, very aggressive GC cell line has been used to TRPM2 role in GC migration and invasion. While AGS is one of the most common cell lines for cell signaling and the xenograft modelling of GC, these cells do not encompass the complexity of GC, and alone may not be representative of all GCs (e.g. androgen-dependent vs. -independent). Further investigations are required to characterize TRPM2-mediated invasion in other GC cell lines.

Overall, we demonstrated a drastic impact of TRPM2 downregulation on *in vitro* invasion and *in vivo* xerograph growth. The observed effect probably resulted from the reduced calcium influx and elevated PTEN activity which led to the downregulation of the Akt signaling and reversion of the EMT processes. Given the impact of TRPM2 on GC survival and metastatic capabilities, our research findings suggest TRPM2 as a valuable alternative therapeutic target to improve the diagnostic of GC and TRPM2 inhibition as a potential strategy to improve its treatment.

## Methods

### Cell culture

AGS and HEK-293 cell lines were purchased from ATCC, and cultured in DMEM/F-12 medium supplemented with 10% heat-inactivated Fetal Bovine Serum (FBS; Gibco, Life Technologies, 16000036) and 20 μg/ml penicillin/streptomycin antibiotic (Gibco; Life Technologies, 15070063). Cells were grown at 37 °C and 5% CO_2_ incubator.

### Real-time PCR assay

Cells were lysed with Trizol (Thermofisher scientific, 15596026) and mRNAs were isolated using Invitrogen RNA Purification kit according to the manufacturer’s protocol. For the synthesis of complementary DNA (cDNA), 2 μg of RNA was used according to the Super Script® II First-Strand Synthesis System (Invitrogen). Gene expression was quantified by real-time PCR using the CFX96 touch real-time PCR instrument (BioRad) and gene-specific primers (Table 1). The mRNA expression data were analyzed based on the Livak and Schmittgen’s 2^−ΔΔCT^ method (3-phosphate dehydrogenase (GAPDH) was considered as a reference gene)^57^.

**Table 1:** The list of primers and their sequences.

Gene	Primer	Sequence 5′ to 3′
GAPDH	Forward Reverse	CTGAAGAGCTGCTTCACCAA ATGGTGCTGTCCTTGACAAC
TRPM2	Forward Reverse	AAGTACGTCCGAGTCTCCCA CGGAAAATGCTCTTCAGCCG
E-Cadherin	Forward Reverse	GAAGGTGACAGAGCCTCTGGAT GATCGGTTACCGTGATCAAAATC
N-Cadherin	Forward Reverse	CCTTTCAAACACAGCCACGG TGTTTGGGTCGGTCTGGATG
Snail	Forward Reverse	ACCACTATGCCGCGCTCTT GGTCGTAGGGCTGCTGGAA
Slug	Forward Reverse	CTGGTCAAGAAGCATTTCAACGCC AAAGAGGAGAGAGGCCATTGGGTA
Vimentin	Forward Reverse	TCTACGAGGAGGAGATGCGG GGTCAAGACGTGCCAGAGAC
ZEB1	Forward Reverse	GCACCTGAAGAGGACCAGAG TGCATCTGGTGTTCCATTTT
Twist	Forward Reverse	CGGAGACCTAGATGTCATTGTT CTTCTATCAGAATGCAGAGGTG
MMP1	Forward Reverse	AGCTAGCTCAGGATGACATTGATG GCCGATGGGCTGGACAG
MMP2	Forward Reverse	CAAGGACCGGTTTATTTGGC ATTCCCTGCGAAGAACACAGC
MMP9	Forward Reverse	TGACAGCGACAAGAAGTG CAGTGAAGCGGTACATAGG
Integrin β1	Forward Reverse	TGGCCTTGCATTACTGCTGA TTGCACGGGCAGTACTCATT
Integrin β3	Forward Reverse	CGAGTGCCTCTGTGGTCAAT TAAAGGTGCAGGCATCTGGG
Integrin β5	Forward Reverse	GTGGGGGTCACCTACAACTG CACAGGTTCTGGTACACGCT
Integrin αv	Forward Reverse	CCAAACTCGCCAGGTGGTAT GCTCCCAGTTTGGAATCGGA
Integrin α5	Forward Reverse	CTATGAGGCTGAGCTTCGGG GGAGAGCCGAAAGGAAACCA

### Western blotting analysis

1x RIPA buffer (20 mM Tris-HCl (pH 7.5), 150 mM NaCl, 1 mM Na2- EDTA, 1 mM EGTA, 1% NP-40, 1% sodium deoxycholate, 2.5 mM sodium pyrophosphate, 1 mM β-glycerophosphate, 1 mM Na3VO4, 1 µg/ml leupeptin) was used to lyse cells and extract cellular proteins. The protein concentration was quantified using the BCA assay protocol (Thermofisher Scientific). For western blot analysis, 20 μg of each protein sample was separated using SDS-gel electrophoresis and transferred onto a 0.45 µm nitrocellulose membrane (BioRad). Membranes were incubated in blocking buffer (5% milk powder dissolved in 1x TBST (137 mM NaCl, 2.7 mM KCl, 19 mM Tris-base, 0.1% TWEEN 20)) for 1 hr at room temperature. The membranes were washed 3 times with 1x PBS and incubated with primary antibodies overnight in 4 °C (The list of primary antibodies is provided in Table 2). A day after, blots were washed with 1x PBS and incubated with the secondary antibody (1:5000; Goat-Anti-Mouse, Goat-Anti-Rabbit; Mandel Scientific) on the shaker for 1 hour at room temperature. Li-Cor Odyssey 9120 infrared imager was used to scan membranes. The band’s intensities were quantified by ImageJ 1.48 v software.

**Table 2:** The list of antibodies.

	Protein name	MW (kDa)	Antibody REF #	Company
1	TRPM2	109, 191	A300–414A-2	BETHYL
2	AKT	60	sc-46915	Santa Cruz Biotechnology
3	phospho-AKT	60	9271 s	Cell Signaling
4	phospho-AKT	60	4058 s	Cell Signaling
5	GAPDH	37	sc-365062	Santa Cruz Biotechnology
6	β-Actin	45	3700 s	Cell Signaling
7	Integrin β1	115, 135	9699 s	Cell Signaling
8	Integrin αv	135, 140	4711 s	Cell Signaling
9	Integrin α5	150	4705 s	Cell Signaling
10	pPTEN	54	9549 T	Cell Signaling
11	PTEN	54	9188 T	Cell Signaling

### Construction and infection of lentiviral vectors to knockdown TRPM2 expression

TRPM2-specific pLKO-shRNA clones were purchased from Dharmacon (TRCN0000044152: AAGTAGGAGAGGATGTTCAGG, TRCN0000044154: ATCCTCATCCAGTATGTACTC). The pLKO.sh.hSC plasmid (Addgene 46896: GAGGGCCTATTTCCCATGATT) was used as the scrambled control. Transfection was performed according to the 3^rd^ generation lentiviral packaging system protocol^58^. Briefly, PPAX2 (6 μg), MD2G (3 μg) and pLKO-LV-gene-specific (6 μg) plasmids were co-transfected into the HEK-293 cells in the presence of PEI transfection reagent (Sigma). The generated lentiviruses were collected and filtered (Millex-GS; 0.22 μm sterile filter) 24 and 48 hrs post-transfection (the prepared virus can be used right away or stored at −80 °C).

A day before transduction, 0.2 million of AGS cells were seeded in 6-well plates and grown for 24 hours. On the day of the experiment, cells medium was replaced with 2 mL of complete cell medium, containing 500 μL of lentivirus aliquot and 8 μg/mL of Sequebrene (Sigma). Seventy-two hours after transduction, the Puromycin-based selection was performed with 1 μg/mL puromycin diluted in complete cell medium (concentration varied based on the cell type) was used to select transduced cells. RT-qPCR and western blot analyses were performed to validate the efficiency of Knockdown.

### Calcium imaging analysis

TRPM2-mediated calcium entry was investigated by calcium imaging analysis using the MetaFlour Olympus analyzer as previously described^59^. Briefly, AGS control and TRPM2-KD cells were seeded in 35 mm bottom-slide plates (MatTek) and grown for 48 hours at 37 °C and 5% CO_2_ incubator. An hour before calcium imaging experiment, cells were incubated with 3 µg/mL Fura-2AM diluted in 1x HBSS for 1 h at room temperature. TRPM2-mediated intracellular calcium elevation was recorded in response to the perfusion of bath solution containing 1 mM H_2_O_2_. Data analysis was done using SigmaPlot12.3.

### Gap closure or wound healing assay

The motility of AGS cells in 2D culture was examined by gap closure assay. Cells were seeded in 2-well culture inserts placed in µ-Dish 35 mm (ibidi). A day after, cells were treated with 10 μg/mL mitomycin (cell proliferation inhibitor) and incubated at 37 °C in 5% CO_2_ incubator for 2 hrs. The mitomycin containing medium was replaced with a complete cell medium, and cells were allowed to recover in a 37 °C incubator for 6 hrs. Later, the inserts were removed, and the first microscopic picture was captured with a conventional 10x phase-contrast objective lens. The gaps were photographed every 6 hrs until they have been filled in control cells. The percentage of gap-filled area in knockdown cells was calculated using the ImageJ software and normalized to control cells. Results were plotted in SigmaPlot12.3 software.

### Cell migration and invasion assay

Cell migration and invasion assays were done using the A3BP48 Three-Tiered Chemotaxis Chamber (Neuro Probe). Cells were starved in FBS free medium 24 hours prior to the experiment. The lower chamber was filled with 25 μL of complete cell-specific medium containing 10% FBS (0% FBS medium was loaded in the negative control wells) and covered by 25 × 80 mm polycarbonate filters (8 μm pores). Fifty μL of the diluted cells (1 million cells per mL in 0% FBS medium) was loaded in each well of the upper chamber. The chamber was wrapped with aluminum foil and incubated at 37 °C and 5% CO_2_ overnight. Next day, the unmigrated cells were removed from the top of the membrane by scraping followed by a wash with 1x PBS. The membrane was stained using the Diff-Quik ^TM^ Stain kit (Siemens) and mounted on the slide using a mounting buffer (Fisher chemical) and covered by a coverslip. Slides were imaged with a conventional 20x phase-contrast objective lens and analyzed using ImageJ software. Invasion assay was differentiated from migration assay by coating the polycarbonate filters with 0.1% gelatin protein a day before the experiment.

### Animal study

To determine the tumor formation ability of TRPM2-KD1 cells in comparison to AGS ShRNA scramble cells, 4 million of cells were subcutaneously injected to the left flank of male SCID mice; 6 mice injected with TRPM2 KD1 cells and 6 mice were injected with ShRNA scramble cells. Two weeks post-injection tumors size were started to measure every three days for around 1.5 months. The tumor-bearing mice were sacrificed at the end of the experiment, and tumors were extracted. Tumour growth rate and the final tumour volume and weight were calculated, and data were plotted using GraphPad Prism 6 software. Animal Protocol was approved by the Dalhousie University committee on laboratory animals (16–137) and all methods and protocols were performed in accordance with Dalhousie institutional guidelines.

### Reagents

Cell culture mediums, FBS, PBS, HBSS, and penicillin/streptomycin antibiotic were purchased from Invitrogen/Thermofisher scientific. LY294002 (PI3K inhibitor) and SC79 (Akt activator) were bought from Sigma-Aldrich.

### Statistical analysis

All experiments were performed at least three times and one biological replicate was represented in each figure. Statistical significance against the control samples (Student’s *t*-test) was calculated in GraphPad Prism 5.0 (Asterisks represent the degree of significance: n.s = p ≥ 0.05, * p = 0.01 to 0.05, **p = 0.001 to 0.01, ***p < 0.001).

## Supplementary information

Original Western Blots
