# Supplementary material for: TRPM2 ion channel promotes gastric cancer migration, invasion and tumor growth through the AKT signaling pathway
Source: Sci Rep. 2019 Mar 12;9:4182. doi: 10.1038/s41598-019-40330-1 (PMC6414629; doi:10.1038/s41598-019-40330-1)
Supplement: Supplementary file 1 — Original Western Blots [file 41598_2019_40330_MOESM1_ESM.pdf]

TRPM2 ion channel promotes gastric cancer migration, invasion and tumor growth through the AKT signaling pathway.

Shekoufeh Almasi[1], Andra M Sterea[2], Wasundara Fernando[3], Derek R Clements[4], Paola Marcato[3,5], David W Hoskin[3,5], Shashi Gujar[3,5], Yassine El Hiani[2] *

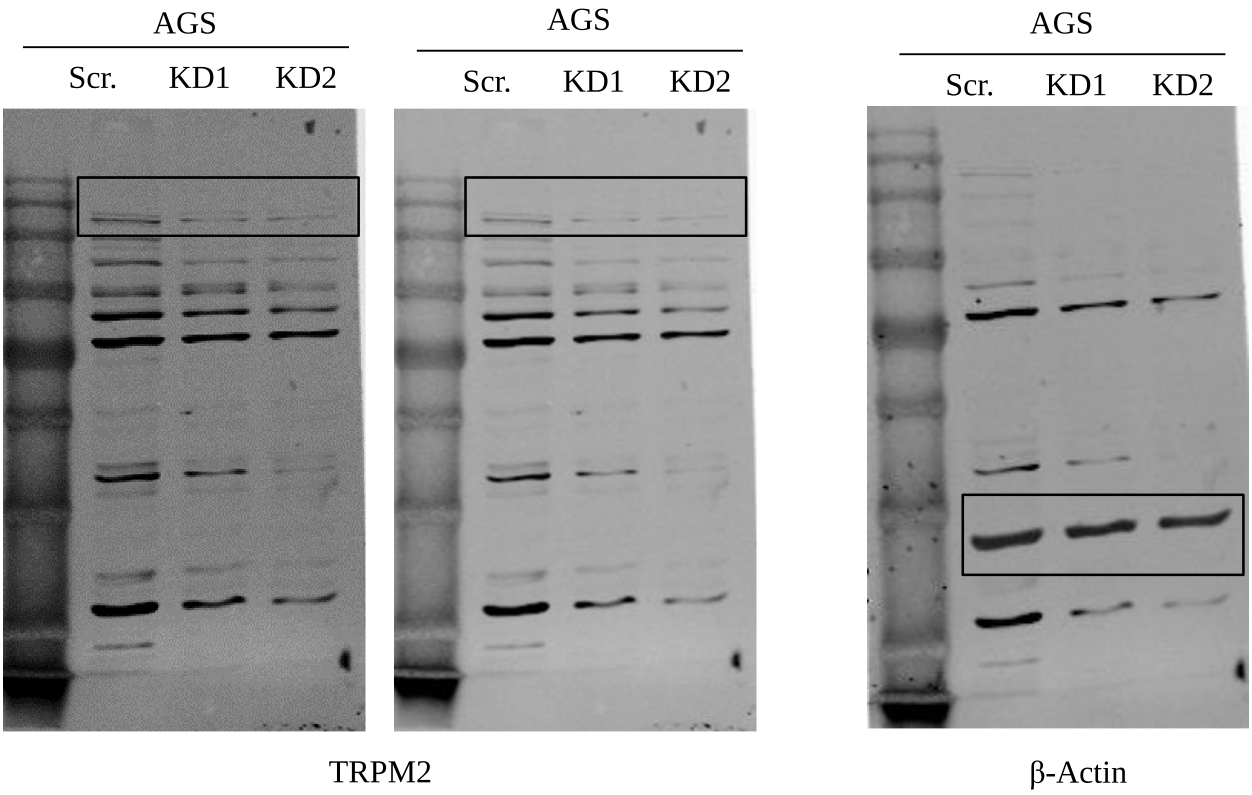

Supplementary Figure 1. Full length immune blots of different exposures with molecular weigh standards for Figure **1A**.

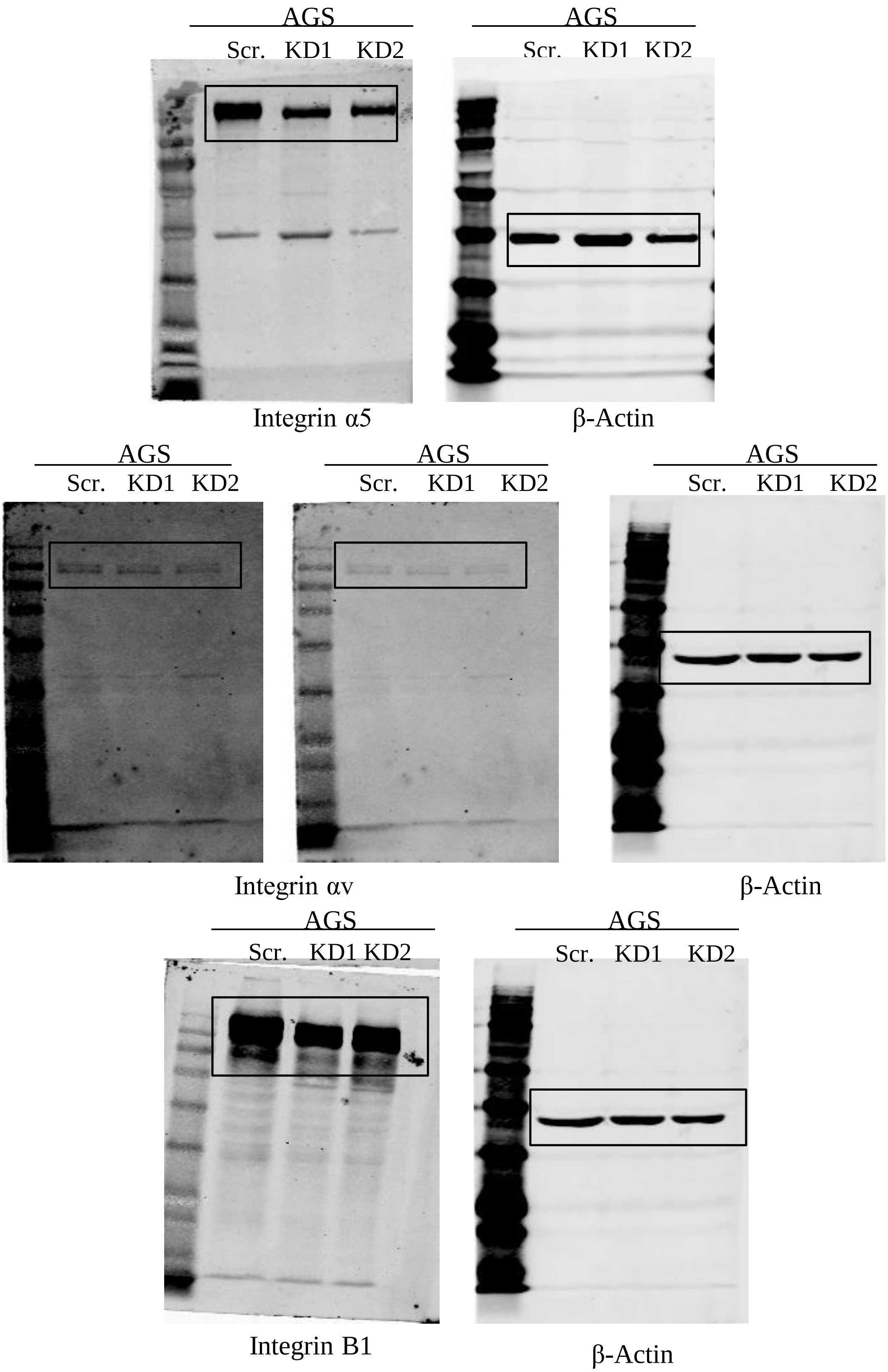

Supplementary Figure 2. Full length immune blots of different exposures with molecular weigh standards for Figure **3C**.

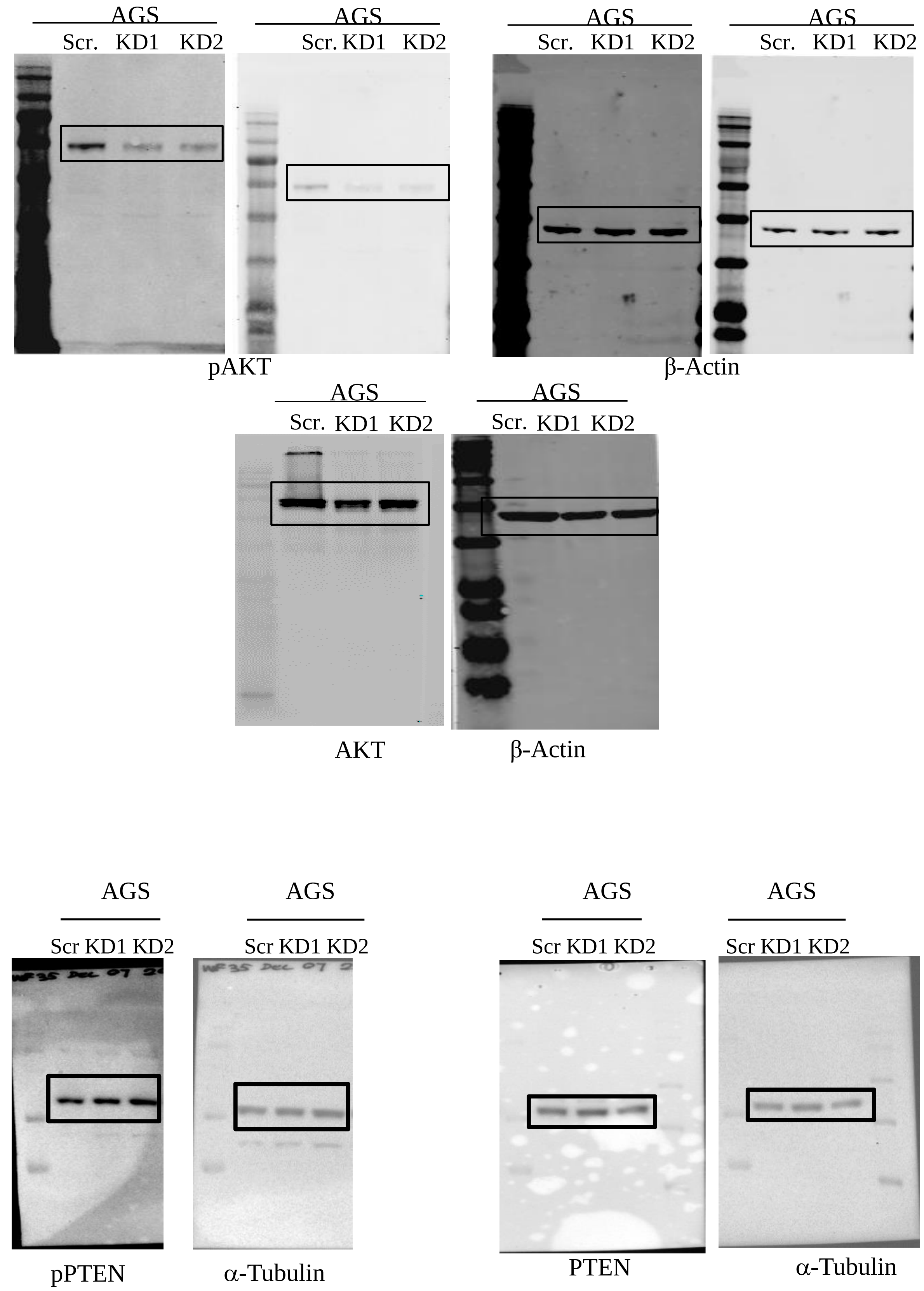

Supplementary Figure 4. Full length immune blots of different exposures with molecular weigh standards for Figure **4A**.

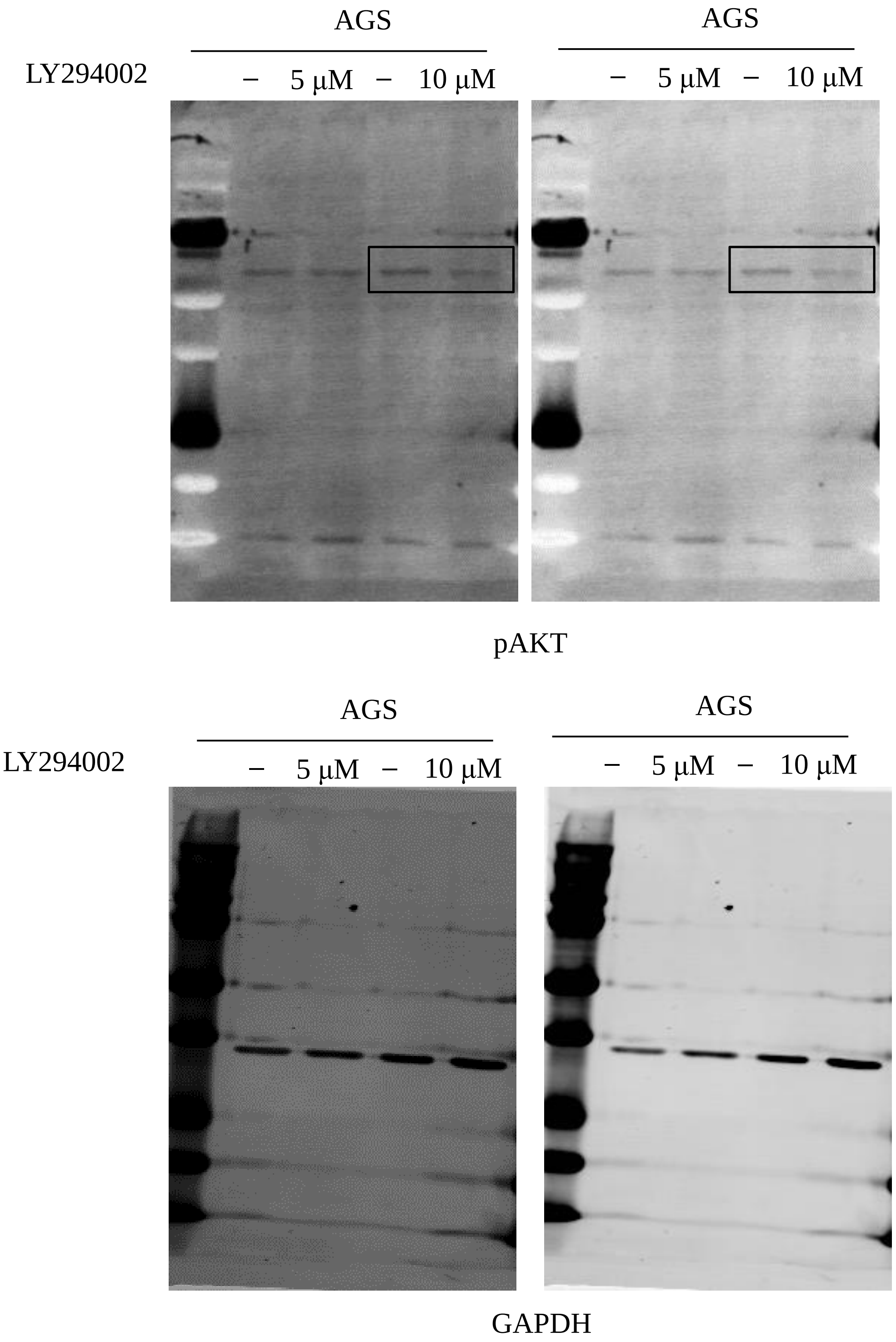

Supplementary Figure 4. Full length immune blots of different exposures with molecular weigh standards for Figure **4B**.

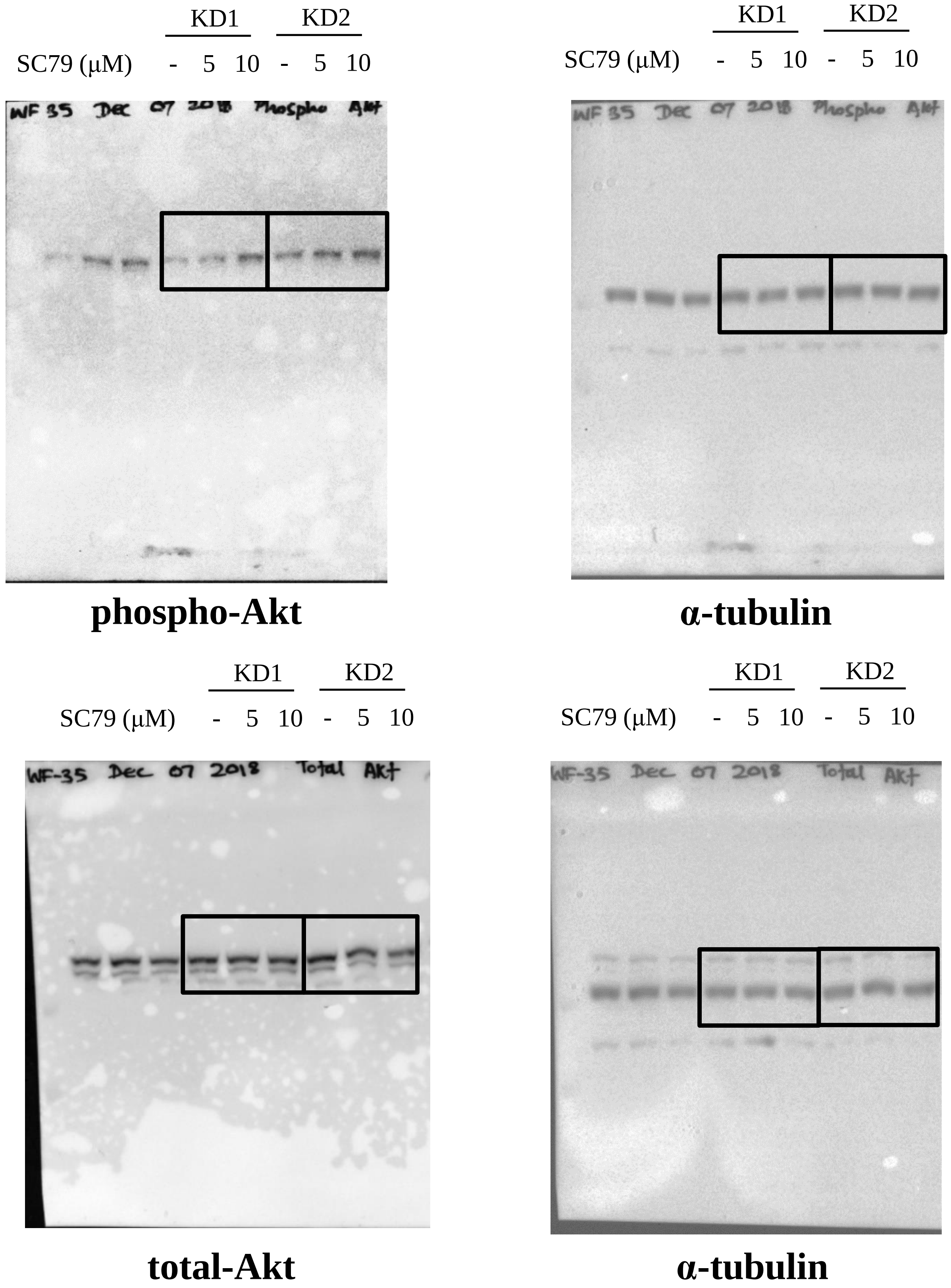

Supplementary Figure 5. Full length immune blots of different exposures with molecular weigh standards for Figure **5A**.
